# Respiratory syncytial virus among hospitalized patients of severe acute respiratory infection in Bhutan: Cross‐sectional study

**DOI:** 10.1111/irv.13242

**Published:** 2024-01-17

**Authors:** Kunzang Dorji, Pema Yuden, Tara Devi Ghishing, Govinda Ghimeray, Chonticha Klungthong, Sonam Wangchuk, Aaron Farmer

**Affiliations:** ^1^ National Influenza Centre, Royal Centers for Disease Control Ministry of Health Thimphu Bhutan; ^2^ ICT Unit Royal Centers for Disease Control, Ministry of Health Thimphu Bhutan; ^3^ Royal Centers for Disease Control, Ministry of Health Thimphu Bhutan; ^4^ Department of Virology Armed Forces Research Institute of Medical Sciences Bangkok Thailand

**Keywords:** adenovirus, human metapneumovirus, influenza virus, respiratory syncytial virus, respiratory tract infection

## Abstract

**Introduction:**

Respiratory syncytial virus (RSV) is a leading cause of lower respiratory tract infections worldwide, particularly in young children. In Bhutan, respiratory disease continues to be among the top 10 diseases of morbidity for several years. This study aimed to estimate the prevalence of RSV among hospitalized patients with severe acute respiratory infection (SARI) in Bhutan.

**Method:**

Respiratory specimens were collected from SARI patients of all ages in 2016 and 2018 following influenza surveillance guidelines. Specimens were tested for influenza and RSV, human metapneumovirus, adenovirus, and human parainfluenza virus types 1, 2, and 3 using real‐time reverse‐transcription polymerase chain reaction assay. Descriptive statistics were used to analyze the result in STATA 16.1.

**Result:**

Of the 1339 SARI specimens tested, 34.8% were positive for at least one viral pathogen. RSV was detected in 18.5% of SARI cases, followed by influenza in 13.4% and other respiratory viruses in 3%. The median age of SARI cases was 3 (IQR: 0.8–21 years) years. RSV detection was higher among children aged 0–6 (Adj OR: 3.03; 95% CI: 1.7–5.39) and 7–23 months (Adj OR: 3.01; 95% CI: 1.77–5.12) compared with the children aged 5–15 years. RSV was also associated with breathing difficulty (Adj OR: 1.73; 95% CI: 1.17–2.56) and pre‐existing lung disease, including asthma (Adj OR: 2.78; 95% CI: 0.99–7.8).

**Conclusion:**

Respiratory viruses were detected in a substantial proportion of SARI hospitalizations in Bhutan.

## 1 INTRODUCTION

Every year, millions of people around the world suffer from severe respiratory infections caused by a respiratory syncytial virus (RSV). This virus, first discovered in chimpanzees, belongs to the genus Orthopneumovirus within the family Pneumoviridae and order mononegavirales.^1^, ^2^

RSV is a leading cause of severe respiratory infection causing hospitalization and death across all age groups, particularly among young children and older adults worldwide. Studies demonstrate that RSV‐associated acute lower respiratory infection (RSV‐ALRI) diseases cause about 64 million cases, 3.4 million hospitalizations, and 118,600 deaths in children aged 5 years and younger.^3^, ^4^, ^5^ Likewise, it is estimated that RSV causes about 1.5 million hospitalizations and 14,000 deaths annually among older adults in industrialized countries.^6^, ^7^ More than 95% of RSV‐ALRI episodes and more than 97% of RSV‐attributable deaths across all age bands were in low‐income and middle‐income countries (LMICs).^8^

Despite the significant impact, limited information is available about the prevalence and epidemiology of RSV infections in Bhutan, a country where respiratory diseases are among the top 10 public health concerns.^9^, ^10^, ^11^ Studies have shown that RSV infections have a seasonal pattern, with peaks occurring during winter months in temperate regions and during rainy seasons in tropical regions.^12^, ^13^ However, there is little or no data on the seasonality of RSV in Bhutan, which has a diverse climate ranging from subtropical to alpine.^14^ Moreover, there is a lack of information on the demographic and clinical characteristics of RSV patients in Bhutan, such as age, gender, comorbidities, symptoms, and outcomes.^15^

Understanding the burden and risk factors of RSV infections in Bhutan is essential for developing effective prevention and control strategies, especially in light of emerging RSV vaccines currently under development, and with some vaccines recently approved for use in elderly adults above 60 years and older.^16^, ^17^, ^18^, ^19^, ^20^, ^21^, ^22^ Therefore, this study aims to determine RSV incidence among SARI cases, seasonal trends, risk factors, and outcomes for patients. Thus, the primary objective is to assess RSV association with demographic and clinical characteristics and to provide valuable information to inform public health strategies and clinical management of SARI in Bhutan.

## 2 METHOD

### 2.1 Study design and setting

**FIGURE 1:**
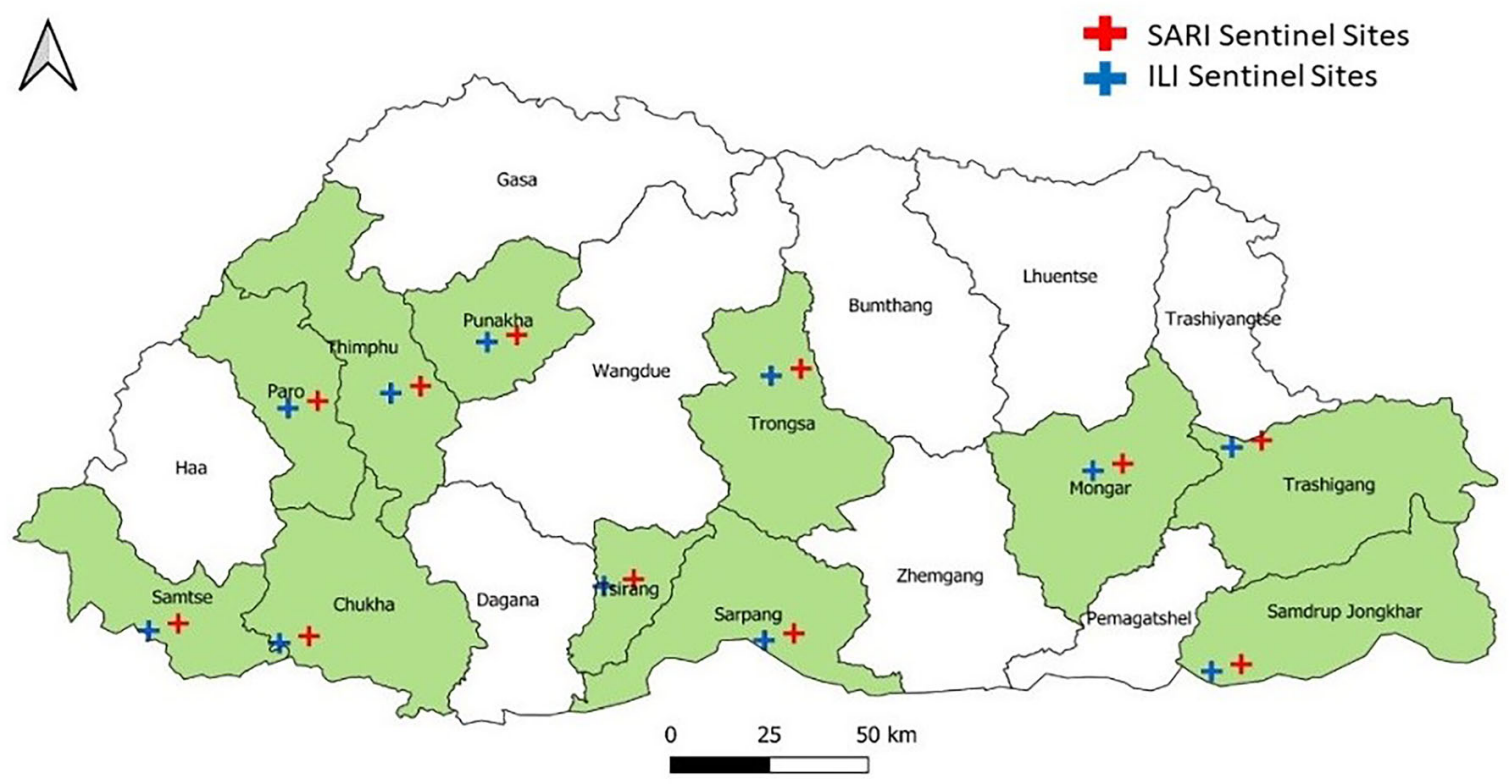
ILI and SARI sentinel surveillance hospitals.

We have adopted a prospective protocol design on existing national ILI and SARI sentinel‐based surveillance for RSV pathogens as per the surveillance guidelines.^23^ Sentinel surveillance hospitals are Jigme Dorji Wanghck National Referral Hospital (JDWNRH), Thimphu, Punakha Hospital, Paro Hospital, Mongar Regional Referral Hospital, Phuentsholing Hospital, Chukha, Gelephu Regional Referral Hospital, Sarpang, Trongse Hospital, Tsirang Hospital, Trashigang Hospital, Samtse Hospital, and Samdrup Jongkhar Hospital (Figure 1). The sentinel sites were selected based on strategic geographical locations and demographic representation.

### 2.2 Study participants

Patients of all age groups hospitalized with SARI were included for respiratory specimen collection in the years 2016 and 2018 as per the SARI case definition: acute respiratory infection with a history of fever or measured fever of ≥38°C and cough with onset within the last 10 days and requiring hospitalization as prescribed in the ILI and SARI surveillance guidelines.^23^, ^24^ Nasal/throat swab specimens for molecular RT‐PCR assays were collected as part of routine patient care through a surveillance network of the Inpatient Department or from the Hospital Emergency Department.

### 2.3 Laboratory detection

#### 2.3.1 Testing algorithm

**FIGURE 2:**
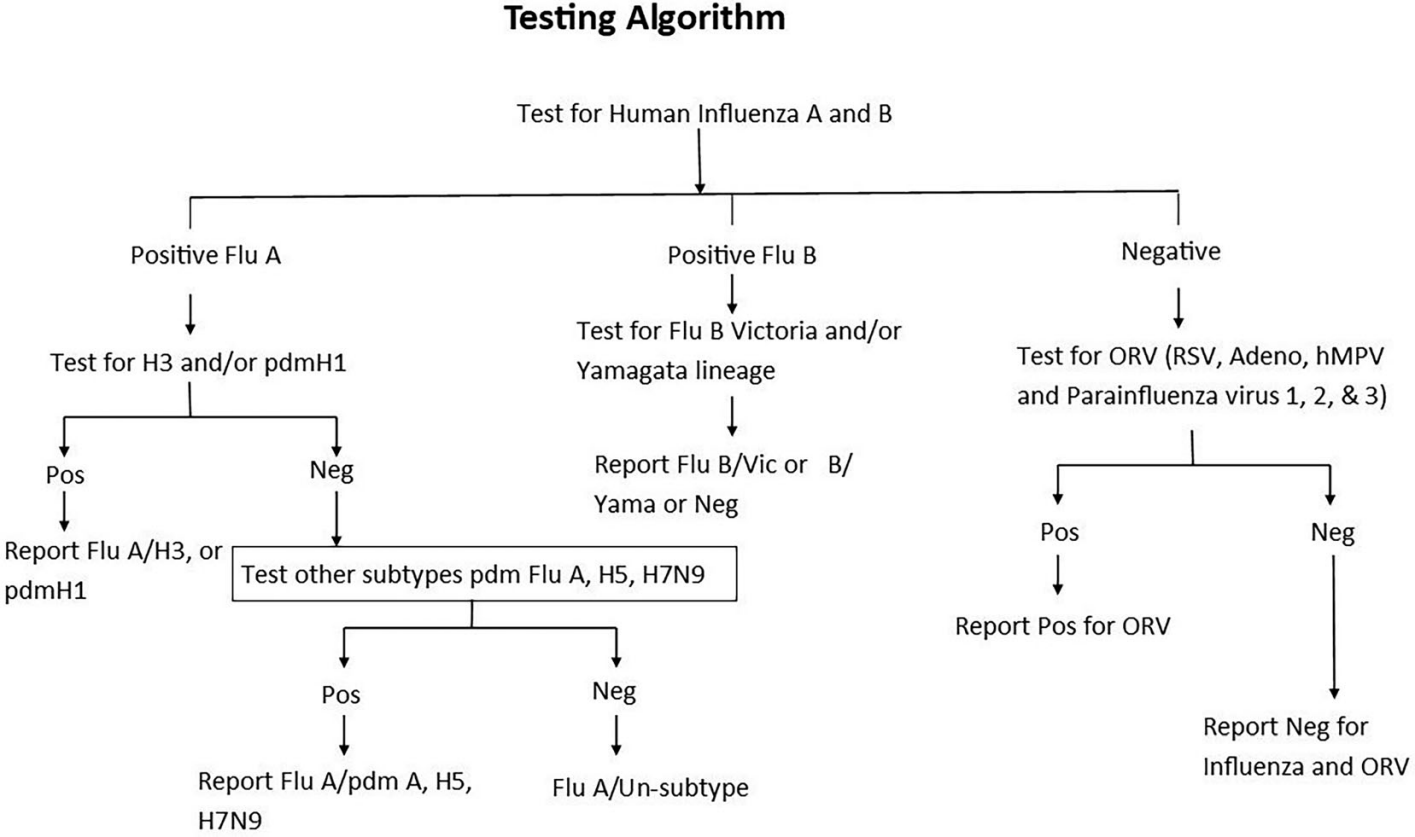
Algorithm for testing the specimen collected.

The SARI specimens were received at the PCR lab in the Royal Centre for Disease Control (RCDC) from the SARI sentinel hospitals every week. The samples were first tested for influenza A and B by real‐time RT‐PCR. All influenza negative samples were further tested for other respiratory viruses (ORV) including RSV, hMPV, adenovirus, and parainfluenza 1, 2, and 3 as prescribed in the testing algorithm below (Figure 2).

#### 2.3.2 RT‐PCR

Viral RNA was extracted from 140 μL of viral transport media containing throat/nasal swabs using a QIAamp viral RNA mini kit (QIAGEN, Germany) following the manufacturer's instructions. The extracted viral RNA was subjected to RSV RT‐PCR Assay. For RT‐PCR reaction, a 25 μL reaction mixture was prepared. This mixture consisted of 5 μL of extracted viral RNA, 5.0 μL of nuclease‐free water, 12.5 μL of 2× Reaction Mix, 1.5 μL forward/reverse primer (40 μM) and 0.5 μL Probe (10 μM), 0.5 μL RnaseOut (10 U/μL), and 0.5 μL SuperScript III RT/Platinum Taq Mix.

The cycling conditions were as follows: a single cycle of reverse‐transcription step at 50°C for 30 min, followed by an initial denaturation step at 95°C for 2 min. Subsequently, 45 amplification cycles were performed, consisting of denaturation at 95°C for 15 s and annealing/extension at 55°C for 30 s. Ct values were used to interpret the results. Ct values of <40 for influenza and RSV, and <35 for the human RNase P (RP) reaction, were considered positive, whereas Ct values of ≥40 for influenza and RSV, and ≥35 for RP, were considered negative. For each protocol, positive and negative controls were run in each test to validate the test result.

### 2.4 Data collection

**FIGURE A1:**
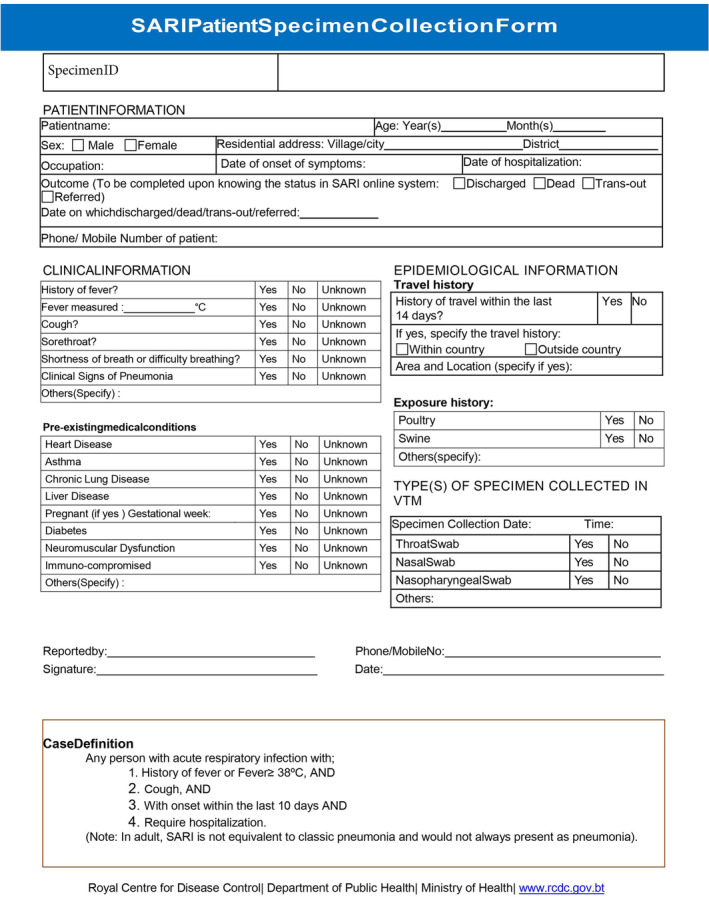
SARI Specimen Collection Form.

Demographic data including clinical and laboratory information were collected through a structured surveillance questionnaire of the SARI Specimen Collection Form (Figure A1), and information on the form was verified before entering into the surveillance system. All laboratory results for influenza and other respiratory viruses were entered into the system, and feedback was shared weekly (FluView Report) with all relevant stakeholders and sentinel sites.^25^

### 2.5 Data analysis

Descriptive statistics were used to analyze the proportion of RSV, influenza, and ORV‐associated SARI in different subgroups. Multivariable logistic regression adjusting for age and sex was used to assess the association of RSV with demographic and clinical characteristics. All data were analyzed using STATA 16.1. QGIS 3.16 was used to generate the mapping distribution of the RSV and influenza positivity in the country.

## 3 RESULT

A total of 1339 SARI cases were enrolled. The median age of SARI was 3 years (IQR: 0.8–21 years) and 56% were males. Virus detection was reported in 34.8% of tested specimens. RSV was detected in 18.5% (248/1339) of SARI cases, followed by influenza in 13.4% (180/1339) and ORV in 2.8% (38/1339). Demographic and clinical characteristics of RSV, influenza, and ORV‐associated SARI cases are shown in Table 1. Among RSV‐associated SARI cases, 86% were below 5 years of age and 52% were females. Among influenza‐associated SARI cases, 45.6% were below 5 years and 61% were males. Clinically, difficulty breathing was more prevalent in RSV‐associated SARI (85%). ICU admission was observed in 6.6% of all enrolled cases and more common in RSV cases (8.2%) as compared with influenza and other viruses (Table 1).

**TABLE 1:** Characteristics of SARI patients admitted to sentinel hospitals in Bhutan in 2016 and 2018.

	All SARI n	Influenza *n* (%)	RSV *n* (%)	Other viruses *n* (%)
	1339	180	248	38
Age group				
0–6 months	182 (13.6)	12 (6.7)	55 (22.2)	2 (5.3)
7–23 months	369 (27.6)	27 (15)	111 (44.8)	22 (57.9)
24–59 months	218 (16.3)	43 (23.9)	46 (18.5)	6 (15.8)
5 to 15 years	152 (11.4)	38 (21.1)	19 (7.7)	1 (2.6)
16–59 years	285 (21.3)	42 (23.3)	9 (3.6)	5 (13.2)
≥60 years	133 (9.9)	18 (10)	8 (3.2)	2 (5.3)
Sex				
Female	583 (43.5)	85 (47.2)	97 (39.1)	18 (47.4)
Male	756 (56.5)	95 (52.8)	151 (60.9)	20 (52.6)
Year				
2016	743 (55.5)	129 (71.7)	142 (57.3)	5 (13.2)
2018	596 (44.5)	51 (28.3)	106 (42.7)	33 (86.8)
Clinical symptoms				
Fever	1174 (87.7)	170 (94.4)	218 (87.9)	35 (92.1)
Cough	1280 (95.6)	174 (96.7)	243 (98)	38 (100)
Sore throat	922 (68.9)	141 (78.3)	170 (68.5)	25 (65.8)
Breathing problem	1021 (76.3)	139 (77.2)	211 (85.1)	28 (73.7)
Comorbidities				
Heart disease	39 (2.9)	7 (3.9)	3 (1.2)	4 (10.5)
Lung condition	38 (2.8)	4 (2.2)	6 (2.4)	3 (7.9)
Liver disease	23 (1.7)	2 (1.1)	2 (0.8)	1 (2.6)
Diabetes	10 (0.7)	0 (0)	1 (0.4)	1 (2.6)
Neuromuscular dysfunction	9 (0.7)	2 (1.1)	1 (0.4)	1 (2.6)
Immunocompromised	14 (1)	4 (2.2)	1 (0.4)	0 (0)
Care seeking				
ICU admission	88 (6.6)	5 (2.8)	20 (8.1)	2 (5.3)
Onset to hospitalization (median days and IQR)	2 (1–4)	2 (1–3.5)	2 (1–4)	2 (1–4)
Onset to sample collection (median days and IQR)	4 (2–6)	4 (2–5)	4 (2–6)	4 (3–6)

**FIGURE 3:**
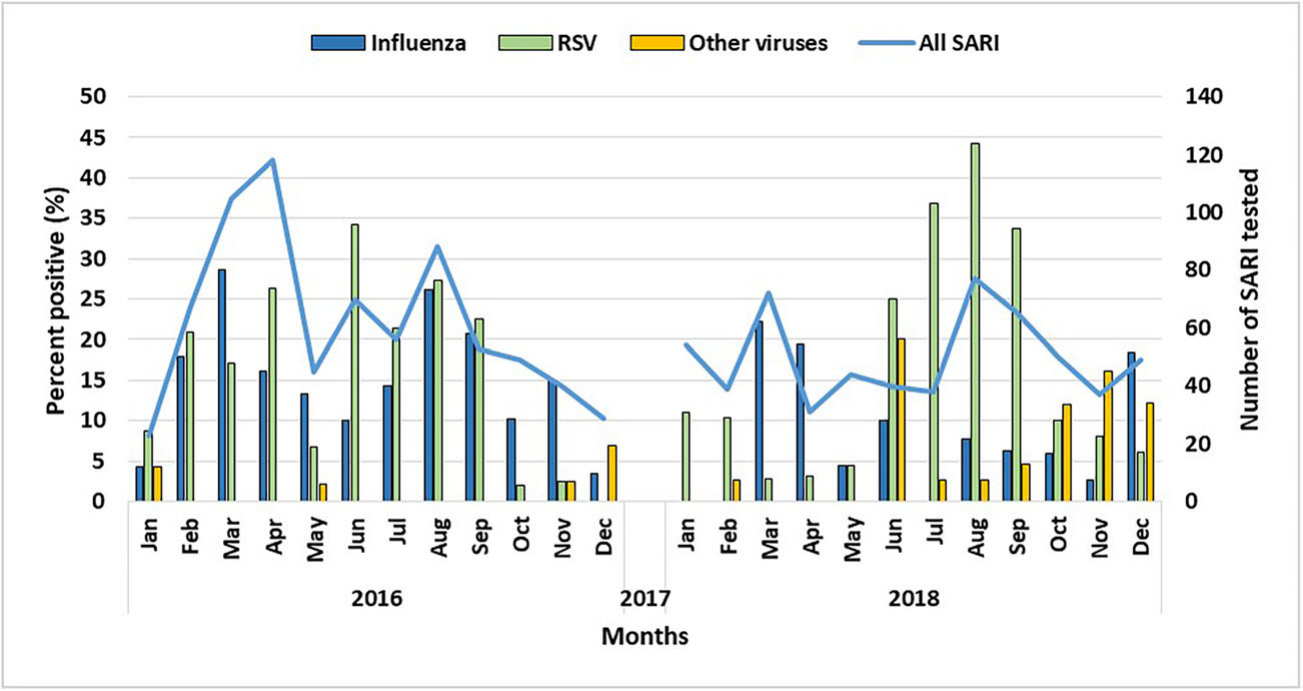
Monthly distribution of viral detection among SARI cases in the year 2016 and 2018.

**FIGURE 4:**
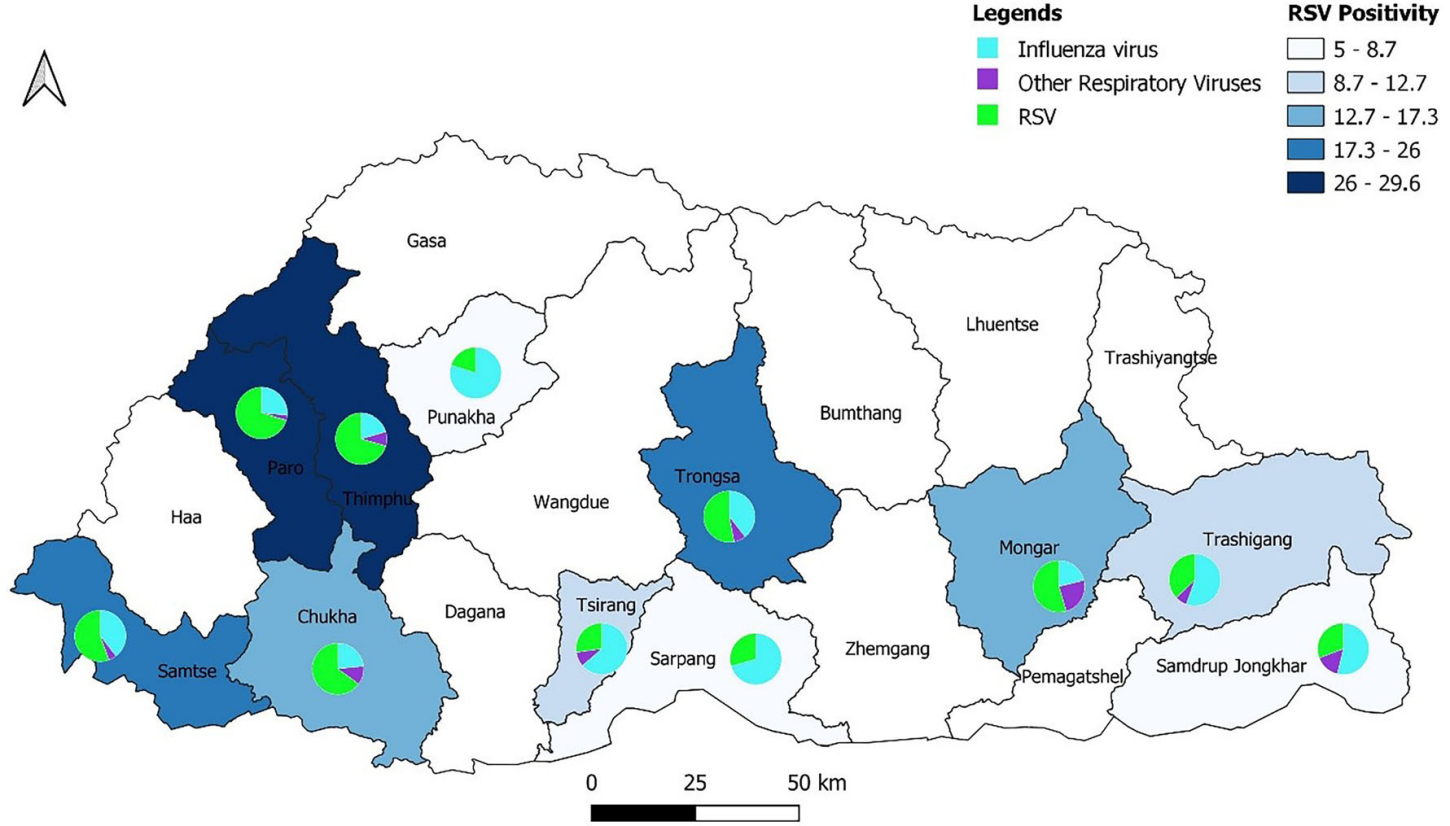
RSV positivity compared with influenza and other respiratory viruses in sentinel sites for all participants.

RSV detection was seen throughout the year in 2016 and 2018 but had an observed in increase positivity rate (>10%) between January to April and June to September in both years (Figure 3). RSV and influenza were detected from all the SARI sentinel hospitals, though RSV positivity was high in JDWNRH, Thimphu, and Paro Hospital with 26% to 29.6% followed by Trongsa Hospital and Samtse Hospital with 17.3% to 26.0% (Figure 4).

RSV detection in SARI cases was more among children aged 0–6 (Adj OR: 3.03; 95% CI: 1.7–5.39), 7–23 (Adj OR: 3.01; 95% CI: 1.77–5.12), and 24–59 months (Adj OR: 1.87; 95% CI: 1.05–3.34) as compared with children aged 5–15 years. RSV was associated with breathing difficulty (Adj OR: 1.73; 95% CI: 1.17–2.56) However, the great majority of infants/children with RSV do not have lung disease (Table 2).

**TABLE 2:** Demographic and clinical characteristics of severe acute respiratory infection positive for RSV compared with those negative for RSV in 2016 and 2018.

	RSV positive *n* (%)	RSV negative *n* (%)	Adjusted OR (95% CI)
All SARI	1091	248	
Age group			
0–6 months	55 (22.2)	127 (11.6)	3.03 (1.7–5.39)
7–23 months	111 (44.8)	258 (23.6)	3.01 (1.77–5.12)
24–59 months	46 (18.5)	172 (15.8)	1.87 (1.05–3.34)
5–15 years	19 (7.7)	133 (12.2)	Ref
16–59 years	9 (3.6)	276 (25.3)	0.23 (0.1–0.52)
≥60 years	8 (3.2)	125 (11.5)	0.45 (0.19–1.06)
Sex			
Female	97 (39.1)	486 (44.5)	0.99 (0.74–1.34)
Male	151 (60.9)	605 (55.5)	Ref
Clinical symptoms			
Fever	218 (87.9)	956 (87.6)	1.12 (0.71–1.74)
Cough	243 (98)	1037 (95.1)	2.24 (0.86–5.85)
Sore throat	170 (68.5)	752 (68.9)	1.24 (0.91–1.69)
Difficulty breathing	211 (85.1)	810 (74.2)	1.73 (1.17–2.56)
Comorbidities			
Heart disease	3 (1.2)	36 (3.3)	0.29 (0.09–0.96)
Lung condition	6 (2.4)	32 (2.9)	2.78 (0.99–7.8)
Liver disease	1 (0.4)	9 (0.8)	0.84 (0.09–7.47)
Diabetes	1 (0.4)	8 (0.7)	0.93 (0.1–8.52)
Neuromuscular dysfunction	1 (0.4)	13 (1.2)	0.39 (0.05–3.15)
Immunocompromised	1 (0.4)	9 (0.8)	0.52 (0.06–4.33)
Care seeking			
ICU admission	20 (8.1)	68 (6.2)	0.96 (0.55–1.69)

## 4 DISCUSSION

In this study, we found respiratory viruses were detected in one‐third of SARI cases, and the most frequently detected virus was RSV followed by influenza. RSV was detected in nearly one in five of all SARI cases. However, we need to consider the SARI case definition, which does not meet the optimal criteria for RSV surveillance as per the WHO; therefore, the case definition needs to be expanded beyond SARI to include cases that do not have a fever or a history of fever. Hospital‐based RSV surveillance will use the extended definition of SARI, also hospital‐based inpatient RSV surveillance in children aged 0–<6 months will additionally include those who present with apnea or sepsis (or both).^26^ Both RSV and influenza circulated throughout the year and had 2 peaks—one between January to April and a second between June to September. However, 2 years of surveillance might also be insufficient to determine seasonality. The same two peak seasons were also reported in influenza activity.^10^

Our study showed that clinically RSV was associated with breathing difficulty and pre‐existing lung disease, including asthma. This is because RSV causes infections of the lungs and respiratory tract. RSV infection can spread to the lower respiratory tract, causing pneumonia or bronchiolitis (inflammation of the small airway passages entering the lungs). It is so common that most children have been infected with the virus by age 2. In adults, RSV symptoms are mild, however, RSV can cause severe infection in some people, including babies 12 months and younger (infants), especially premature infants, older adults, people with heart and lung disease, or anyone with a weak immune system (immunocompromised).^27^

Our findings showed RSV prevalence (18.5%) among SARI patients were more compared with influenza (13.4%) and ORV (2.8%), which is similar to other countries in the Southeast Asian region including India,^28^ Thailand,^29^ Bangladesh,^30^ Indonesia,^31^ and Malaysia^32^; however, one study from India found influenza (12.7%) was detected more than RSV (8.2%) in SARI patients.^33^ According to one study, the reported RSV positivity rates in tropical Asian countries range from 9% to 50% depending on the geographic location and sample population.^34^

In this study, 86% of RSV‐associated SARI admissions were below 5 years of age, and more among children aged 0–6 (Adj OR: 3.03; 95% CI: 1.7–5.39), 7–23 (Adj OR: 3.01; 95% CI: 1.77–5.12), and 24–59 months (Adj OR: 1.87; 95% CI: 1.05–3.34) as compared with children aged 5–15 years. This is in concordance with many studies demonstrating RSV is among the most common causes of lower respiratory tract infections in children below 5 years.^35^, ^36^, ^37^, ^38^ In addition, research studies show preterm infants have a higher risk for RSV infection,^37^ with other risk factors including poor feeding, vomiting, and the rainy season.^32^ Given maternal administration of RSV vaccines during pregnancy are promising strategy to prevent and reduce RSV infection in newborns, the data in this study may support future investigation of these strategies in Bhutan.^39^, ^40^

Improved understanding of RSV seasonality at the national level is important to ensure the optimal timing of prevention and control measures. Our study showed that RSV was observed throughout the year in 2016 and 2018; however, the peak season occurred in the winter season (January to April) and Monsoon season (June to September) in both years, which is consistent with previous studies and WHO findings.^15^, ^41^ These studies also showed there is less consistency in RSV epidemic seasonality across countries experiencing a subtropical climate like Bhutan, Singapore, and Ecuador, compared with the temperate climates of the Northern hemisphere where cases increase every winter.^15^, ^42^, ^43^, ^44^ One study found that RSV seasonality is closely associated with the latitude of the country,^45^ and although Bhutan is a small country that experiences four different seasons^46^ and falls partly in the subtropical climate, it was recently mapped in the Northern hemisphere by the WHO.^47^, ^48^

RSV and influenza were detected from all the SARI sentinel hospitals, but RSV positivity was high in JDWNRH, Thimphu, and Paro Hospital with 26% to 29.6% followed by Trongsa Hospital and Samtse Hospital with 17.3% to 26.0%. As JDWNRH is the only national referral hospital in the country, many people avail medical services including maternal and child health services.^11^ Further, Thimphu being the capital city, around one fourth of the population resides in Thimphu and caters health services to nearby district's self‐referral patients.^49^

### 4.1 Strength and limitation

The strength of this study is we included all age groups while other studies mainly focused on pediatric age groups. However, there are few limitations that affect our result analysis. First, our study has not adopted an extended SARI case definition to capture the RSV cases. Second, outcome status is not known for nearly 40% of participants due to incomplete information during data collection. Third, we did not have additional years of laboratory data of 2017 due to a shortage of supply of laboratory reagents limiting testing capacity. Likewise, RSV typing and gene sequencing were not performed due to a lack of necessary supplies. Lastly, other than medical comorbidities, other risk factors such as preterm and poor feeding were not assessed as the information was not captured by a routine surveillance questionnaire.

## 5 CONCLUSION

Respiratory viruses were detected in a substantial proportion of SARI hospitalizations in Bhutan with RSV detected in nearly one in five of all SARI cases. The high prevalence of RSV in hospitalized SARI infants underscores the importance of RSV prevention, which will soon be available with maternal vaccine or infant monoclonal antibodies (mAb). Therefore, surveillance for RSV is necessary to inform clinical management of SARI, particularly in children, and to implement programmatic preventative and control measures including RSV vaccination.

## AUTHOR CONTRIBUTIONS

All authors have equally made substantial contributions in writing this manuscript as follows: Kunzang Dorji—conception and design of the study, acquisition and curation of the data, analysis and interpretation of the data, drafting of the manuscript, and critical revision; Pema Yuden—testing of the samples; Tara Devi Ghishing and Govinda Ghimeray—data curation and acquisition of the data. Chonticha Klungthong, Sonam Wangchuk, and Aaron Farmer—review and revise it critically for important intellectual content. All authors reviewed the manuscript for the final approval of the version to be submitted.

## CONFLICT OF INTEREST STATEMENT

All authors declared that they have no conflict of any financial interest to disclose.

### PEER REVIEW

The peer review history for this article is available at https://www.webofscience.com/api/gateway/wos/peer-review/10.1111/irv.13242.

## ETHICS STATEMENT

This study was part of an influenza control program evaluation; the protocol is being reviewed and approved annually by the Research Ethics Board of Health (REBH), Ministry of Health, Bhutan. Administrative clearance was obtained from the Institute: Royal Centre for Disease Control. The study used anonymized routine surveillance data; we have maintained and protected patient privacy information.

## DISCLAIMER

Material has been reviewed by the Walter Reed Army Institute of Research. There is no objection to its presentation and/or publication. The opinions or assertions contained herein are the private views of the author and are not to be construed as official or as reflecting true views of the Department of the Army or the Department of Defense. The investigators have adhered to the policies for the protection of human subjects as prescribed in AR 70–25.

## Data Availability

All the data including clinical information and materials will be available on a request basis to the corresponding author.
